# Identification of reproductive performance in Bali-polled bulls using computer-assisted semen analysis and plasma seminal proteomics

**DOI:** 10.14202/vetworld.2025.102-109

**Published:** 2025-01-14

**Authors:** Athhar Manabi Diansyah, Sanstoso Santoso, Herdis Herdis, Muhammad Yusuf, Tri Puji Priyatno, Tulus Maulana, Abdul Latief Toleng, Muhammad Ihsan Andi Dagong, Syahruddin Said, Hikmayani Iskandar, Aeni Nurlatifah, Puji Lestari, Lukman Affandy, Abdullah Baharun

**Affiliations:** 1 Research Center for Animal Husbandry, National Research and Innovation Agency, Cibinong Science Center, Jl. Raya Jakarta, Bogor, West Java 16915, Indonesia; 2 Department of Animal Production, Faculty of Animal Science, Hasanuddin University, Indonesia. Jl. Perintis Kemerdekaan 10 Tamalanrea Makassar, South Sulawesi 90245, Indonesia; 3 Research Center for Applied Zoology, National Research and Innovation Agency, Jl. Raya Bogor Km. 46, Cibinong, West Java 16911, Indonesia; 4 Department of Nutrition and Feed Technology, Faculty of Animal Science, Gadjah Mada University, Depok, Sleman Regency, Special Region of Yogyakarta, Indonesia, 55281; 5 Department of Animal Science, Faculty of Agriculture, Djuanda University, Jl. Tol Ciawi No. 1, Ciawi, West Java 16720, Indonesia

**Keywords:** artificial insemination, Bali-polled bulls, fertility biomarkers, seminal plasma proteomics, sperm kinematics,

## Abstract

**Background and Aim:**

Bali-polled bulls, known for their favorable traits, require advanced reproductive performance analysis to optimize breeding programs. This study aimed to evaluate sperm kinematics and seminal plasma proteomic profiles as biomarkers for sperm motility and fertility in Bali-polled bulls.

**Materials and Methods:**

Semen from five Bali-polled bulls was collected biweekly over five batches using artificial vaginas. Sperm kinematics were assessed using computer-assisted semen analysis (CASA). Fertility was evaluated through service per conception (S/C) in artificial insemination trials. Seminal plasma proteins were analyzed through liquid chromatography–tandem mass spectrometry (LC-MS/MS) and annotated using the UniProt database, PANTHER for gene ontology, and STRING database for protein interactions.

**Results:**

Post-thaw sperm kinematics showed satisfactory results, with a mean S/C of 1.52. Proteomic analysis identified 138 proteins, including six (TEX101, BSP1, PRSS55, BSP3, SPADH2, and TPPP2) linked to sperm motility. These proteins were involved in key biological processes such as sperm capacitation, motility regulation, and sperm-oocyte interaction.

**Conclusion:**

Sperm kinematics and seminal plasma proteomics provide insights into Bali-polled bull fertility. Identified proteins can serve as fertility biomarkers, aiding in superior local breed development and reproductive efficiency improvement.

## INTRODUCTION

The development of local bulls in Indonesia has progressed to produce superior livestock bulls. This development is supported by livestock technology, which plays a vital role in its progress. One of the local cattle that has recently been developed, namely the Bali-polled bull, has the advantages of easy and safe management, better weight and carcass growth, and greater disease resistance [1, 2]. Reproductive success is a critical factor in the development of superior livestock breeds, so the bull’s ability is essential in determining the efficiency and success of this development.

Several methods have been developed to identify the fertility capabilities of bulls, including using computer-assisted semen analysis (CASA), which can evaluate specific sperm cell movement, reduce subjectivity, and obtain more accurate results, thus being able to diagnose the fertility capabilities of bulls [3]. The use of CASA has been widely investigated by observing the kinematics of spermatozoa, which is closely related to their fertilization ability [4, 5]. However, using CASA still requires the combination of several other parameters. Therefore, a more detailed molecular analysis of the protein components related to fertility properties is critical. According to Kaya and Memili [6], male fertility in a breeding program depends not only on the quantity and quality of semen produced. In line with this opinion, Viana *et al*. [7] demonstrated that sperm quality can be affected by several variables, including components of seminal plasma and interpersonal factors involving sperm. In addition, depending on the concentration of protein in the seminal plasma, protein function regulates the fertility of bulls [8]. Identifying protein markers for fertility, including proteomic analysis of seminal plasma, is the current focus of fertility assessments [9]. The quality of spermatozoa is influenced by the interaction and transcription of many proteins [10]. In cattle, the potential function of genomics as a biomarker for determining bull fertility can be determined by identifying seminal plasma content, including plasma proteins. Plasma proteins and other seminal plasma biochemical components are biomarkers of bull seminal plasma [11]. Biochemical components in seminal plasma, including proteins and peptides, regulate fertilization, capacitation, spermatogenesis, and protection of spermatozoa [12]. However, information regarding the sperm cell kinematics and plasma proteomics of Bali-polled bulls is limited.

This study aimed to identify sperm cell kinematics and seminal plasma proteomic profiles as biomarkers of motility in Bali-polled bulls. The novelty of this research lies in its comprehensive analysis of sperm cell kinematics and seminal plasma proteomics in Bali-polled bulls, which is the first of its kind. By identifying specific kinematic parameters and proteomic profiles that serve as biomarkers for sperm motility, this study opens new avenues for developing diagnostic tools to enhance the selection and management of Bali-polled bulls based on semen quality. Furthermore, the findings offer significant insights into the reproductive biology of Bali-polled bulls, which has been previously underexplored. This study could contribute to scientific knowledge and also support the conservation and improvement of local genetic resources, as well as the breeding development of superior Balinese bulls.

## MATERIALS AND METHODS

### Ethical approval

The study was approved by the Animal Ethics Commission of the Faculty of Animal Husbandry, Hasanuddin University.

### Study period and location

The study was conducted from February 2022 to February 2024 at the Laboratory of Animal Reproduction, Semen Processing Unit, Faculty of Animal Science, Hasanuddin University, Makassar, and the Research Center for Applied Zoology, National Research and Innovation Agency, Cibinong, Indonesia.

### Animals

A total of 5 Bali-polled bulls aged 5–10 years were used for the study. Semen was collected twice a week using an artificial vagina. The bulls were maintained according to the standard operating procedures (SOP) of the South Sulawesi Regional Artificial Insemination (AI) Center. Bali-polled bulls were individually housed in 2.5 × 2 m cages equipped with feed and drink containers.

### Semen processing

Before the advanced test, the semen needs to be processed for longer storage. The semen was processed as described by Diansyah *et al*. [4]. Following the AI Center’s SOP for manufacturing frozen sperm using a commercial extender (Andromed, Minitube, Germany) at a ratio of 1:4 with Aquabidest, followed by homogenization with semen at a concentration of sperms per straw of 25 × 10^6^. Furthermore, the semen was equilibrated for 4 h at 5°C. The frozen semen was prepared by pre-freezing by exposing the straw to nitrogen vapor for 10 min. The frozen sperm were placed in a −196°C liquid nitrogen container.

### Post-thawing sperm kinematics

Frozen semen was thawed in a water bath at 37°C for 30 s. The straws were thoroughly dried and evacuated in a pre-warmed Eppendorf tube by cutting the manufactory and laboratory plugs at both ends of the straw. Subsequently, the tube containing the semen was placed in a water bath at 37°C for further evaluation. Sperm kinematics was evaluated by dripping 10 μL of semen on a glass object and covering the object with a glass covering. Sperm kinematics were evaluated using CASA (Vision Version™ 3.7.5 program Minitube, Germany) based on the following: Distance curve linear (DCL), distance average path (DAP), distance straight line (DSL), velocity curve linear (VCL), velocity average path (VAP), velocity straight line (VSL), linearity (LIN), straightness (STR), and wobble (WOB) [13].

### Fertility test

A fertility test was performed with the success rate of AI. Semen from Bali-polled bulls was inseminated in 50 cows aged 3–9 years with parity 2–7 and body condition score 4–7 (Scale 9) without synchronization protocol. All cows were given forage feed, drinking water, and vaccinations. In the fertility test, service per conception (S/C) was measured, as described by Yusuf *et al*. [14]. Different factors that may affect the success rate of AI were not assessed.

### Confirmation of the seminal plasma protein profile

**Figure 1:**
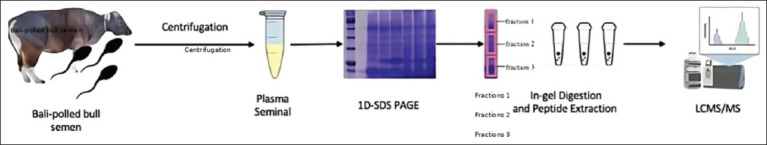
Schematic workflow for confirming the protein profile of Bali-polled seminal plasma.

One-dimensional sodium dodecyl sulfate-polyacrylamide gel electrophoresis (1D-SDS-PAGE) and liquid chromatography–tandem mass spectrometer (LCMS/MS) analysis determined the confirmation of the profiling proteins of seminal plasma (Figure 1).

### 1D-SDS-PAGE

The semen was centrifuged for 30 min at 3000× *g*. The supernatant was stored in a microtube at −20°C following centrifugation. 1D-SDS-PAGE (SMOBIO™ Technology, Inc., Taiwan) followed the procedures of Diansyah *et al*. [15] by first measuring the total dissolved protein concentration of the sample to be identified using the Bradford method with bovine serum albumin (Sigma-Aldrich, USA) as the standard. SDS-PAGE analysis was performed to determine the protein profile based on the molecular weight expressed in the form of bands on the gel. Protein separation was performed using 12% polyacrylamidegel containing SDS and 4% stacking gel with a 60 V voltage current strength of 20 mA (for 3.5 h) [15]. Gel staining was performed using Coomassie Brilliant Blue with the marker from Excel and 3-color Broad Range Protein Marker PM 2700 (Smobio™ Technology, Inc.) with a molecular weight range of ~5–350 kDa. The protein band expressed on the gel band was excised and corresponds to the target protein based on its molecular weight.

### LCMS/MS

The ribbon pieces of the protein bands were placed into a sterile microtube and 200 μL of destaining solution (80 mg ammonium bicarbonate in 20 mL acetonitrile [ACN] and 20 mL ultrapure water) was added. The dye-washing process was carried out for 30 min at 37°C in two washes. The gel pieces were then added with 30 μL reducing buffer (3.3 μL Tris (2 carboxyethyls) phosphine into 300 μL digestion buffer (10 mg ammonium bicarbonate in 5 mL ultrapure water) and incubated (10 min, 60°C). Protein samples were then subjected to alkylation using 30 μL of alkylation buffer (Iodoacetamide in digestion buffer) for 1 h at room temperature. The sample was washed using 200 μL of destaining solution twice (15 min, 37°C). Trypsin digestion was started by adding 50 μL 100% ACN samples for 15 min at room temperature and then freeze-dried (5–10 min). The samples were added with 50-μL digestion buffer and 10 ng/μL activated trypsin (4 h at 37°C). The activated peptide samples were then purified using Thermo Scientific Pierce C 18 Spin Columns (bind peptides) (Thermo Fisher Scientific, Illinois, USA). Peptide clean-up was initiated by activating the resin with 200 μL 50% ACN and centrifuging (1500× *g*, 5 min). The spin column was added with 200 μL of equilibration solution (0.5% trifluoroacetic acid in 5% ACN) and centrifuged (1500× *g*, 5 min). 150 μL of the sample was placed into the spin column and centrifuged again (1500× *g*, 5 min). The spin column was then washed with 200 μL of equilibration solution by centrifugation (1500× *g*, 5 min). The sample was then eluted using 20 μL 70% ACN and centrifuged (1500× *g*, 3 min). The spin column containing the sample is then dried and injected into the LC-MS/MS [16].

### Statistical analysis

The data on sperm kinematics and fertility rates were analyzed using descriptive statistics and presented as means ± standard deviations. Statistical comparisons were conducted using IBM SPSS Statistics version 25 (IBM Corp., Armonk, NY, USA). Analysis of variance (ANOVA) was used to determine significant differences between groups where applicable. *Post hoc* comparisons were performed using Tukey’s test to identify specific group differences. Correlations between sperm kinematic parameters and fertility outcomes were evaluated using Pearson’s correlation coefficient. For proteomic data, the UniProt protein database (http://www.uniprot.org) was utilized for protein identification, and functional annotations were conducted using the PANTHER database (http://pantherdb.org). Protein interaction networks were analyzed through STRING database (http://string-db.org). Statistical significance was set at p < 0.05.

## RESULTS

### Post-thaw sperm kinematics and fertility rate of Bali-polled bulls

The post-thaw sperm kinematics and fertility rate of Bali-polled bulls are presented in Table 1. The parameters measured include distance curve linear (DCL), distance average path (DAP), distance straight line (DSL), velocity curve linear (VCL), velocity average path (VAP), velocity straight line (VSL), linearity (LIN), straightness (STR) and wobble (WOB). The mean ± SD and 95% confidence interval of each parameter are presented in the table. The service/conception rate is also reported as 1.52 ± 0.08, with a 95% confidence interval of 1–3.

**Table 1:** Post-thawing kinematic sperm and fertility rate of Bali-polled bulls.

Parameter	Mean ± SD	Confidence interval, 95%
DCL (mm)	31.19 ± 1.04	29.90–32.48
DAP (mm)	20.80 ± 1.88	18.46–23.13
DSL (mm)	12.20 ± 1.02	10.94–13.46
VCL (mm/s)	111.70 ± 2.93	108.06–115.34
VAP (mm/s)	64.80 ± 3.54	60.41–69.20
VSL (mm/s)	37.86 ± 1.07	31.88–44.34
LIN (%)	34.00 ± 4.64	28.24–39.36
STR (%)	58.60 ± 5.59	51.65–65.55
WOB (%)	58.00 ± 2.74	54.60–61.40
Service/conception	1.52 ± 0.08	1–3

DCL=Distance curve linear, DAP=Distance average path, DSL=Distance straight line, VCL=Velocity curve linear, VAP=Velocity average path, VSL=Velocity straight line, LIN=Linearity, STR=Straightness, WOB=Wobble

### Protein profile of the seminal plasma of Bali bulls

**Figure 2:**
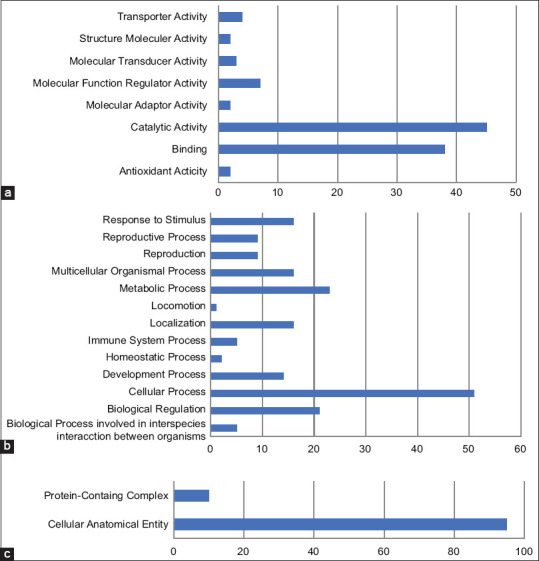
Gene ontology terms of seminal plasma Bali-polled bull based on (a) molecular function, (b) biological process, and (c) cellular component.

A total of 138 genes had functional annotation from Bali-polled bull based on GO, which was analyzed using the PANTHER software because the database had a limited number of proteins from *Bos javanicus*; all of the proteins reported were of *Bos taurus* origin. (Figure 2). The biological processes linked with proteins found in the seminal plasma were antioxidant activity (2 genes), binding (38 genes), catalytic activity (45 genes), molecular adaptor activity (2 genes), molecular function regulator activity (7 genes), molecular transducer activity (3 genes), structure molecular activity (2 genes), and transporter activity (4 genes). The biological process involved in interspecies interaction between organisms (5 genes), biological regulation (21 genes), cellular process (51 genes), development process (14 genes), homeostatic process (2 genes), immune system process (5 genes), localization (16 genes), locomotion (1 gene), metabolic process (23 genes), multicellular organismal process (16 genes), reproduction (9 genes), reproduction process (9 genes), and response to stimulus (16 genes). The cellular components were cellular anatomical entities (95 genes) and protein-containing complexes (10 genes).

**Figure 3:**
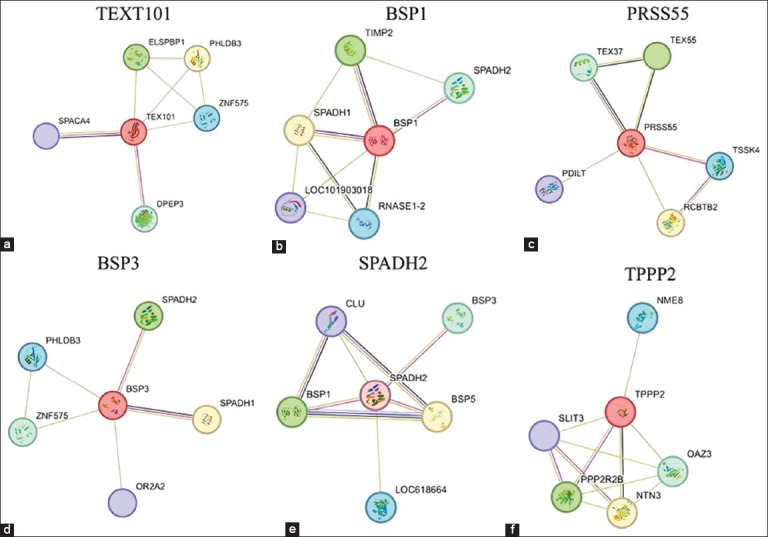
Interaction seminal plasma proteins of Bali-polled bulls associated with sperm motility function: (a) Testis expressed 101, (b) binders of sperm protein 3, (c) Serine protease 55 (protease serine 55), (d) binders of sperm protein 1, (e) sperm adhesion molecule 2, and (f) tubulin polymerization promoting protein family member 2.

The protein-specific seminal plasma in Bali-polled bulls that were found to be associated with sperm motility function included testis expressed 101 (TEX101) (27.3 kDa), binders of sperm protein 1 (BSP1) (16.1 kDa), protease serine 55 (PRSS55) (36.1 kDa), binders of sperm protein 3 (BSP3) (15.5 kDa), sperm adhesion molecule 2 (SPADH2) (13.4 kDa), and tubulin polymerization promoting protein family member 2 (TPPP2) (18.6 kDa) (Table 2). The protein STRING analysis revealed that TEX101 interacted with several seminal plasma proteins: Sperm Acrosome Associated 4, Dipeptidase 3, Zink Finger Protein 575 (ZNF575), Pleckstrin Homology Like Domain Family B Member 3 (PHLDB3), and Epididymal Sperm Binding Protein 1 (Figure 3a). BSP1 interacted with Ribonuclease A Family Member 12, LCO101903018 (Acrosin inhibitor 1), Sperm Adhesion Molecule 1 (SPADH1), Metalloproteinase inhibitor 2, and SPADH2 (Figure-3b). PRSS55 interacted with RCC1 and BTB domain-containing protein 2 (RCBTB2), Protein disulfide isomerase like, testis expressed (PDILT), Testis-expressed sequence 37 protein (TEX37), Testis-expressed sequence 55 protein (TEX55), and Testis specific serine kinase 4 (TSSK4) (Figure-3c). BSP3 interacted with SPADH1, SPADH2, PHLDB3, and ZNF575, and olfactory receptor 2A2 (Figure-3d). SPADH2 interacted with BSP1, clusterin alpha chain, BSP3, binders of sperm protein 5, and LOC618664 (Ecto-ADP-ribosyltransferase 5) (Figure-3e). TPPP2 interacted with thioredoxin domain-containing protein 3 (NME8), slit-like protein 3 protein (SLIT3), serine/threonine-protein phosphatase 2A 55 kDa regulatory subunit B (PPP2R2B), netrin-3 (NTN3), and ornithine decarboxylase antizyme 3 (OAZ3) (Figure-3f).

**Table 2:** Protein-specific seminal plasma levels associated with sperm motility in Bali-polled bulls.

Protein accession	Gene symbol	Protein name	Molecular weight (kDa)
A6QPE3	TEX101	TEX101 protein	27.3
P04557	BSP3	Seminal plasma protein A3	16.1
E1BLW6	PRSS55	Serine protease 55	36.1
P02784	BSP1	Seminal plasma protein PDC-109	15.5
P82292	SPADH2	Spermadhesin Z13	13.4
Q3T077	TPPP2	Tubulin polymerization-promoting protein family member 2	18.6

## DISCUSSION

Sperm kinematic evaluation of semen quality may assist in selecting cryopreserved semen in bulls [3]. CASA provides accurate annotation of spermatozoa movement attributes [17]. The CASA assessment proposed potential clues to accurately estimate bull richness compared with conventional semen assessment strategies [18]. Table 1 shows that the Bali-polled bulls in this study had excellent sperm kinematics. The results of this study indicate that sperm kinematics are likely similar to those of other local bulls reported by Hasan *et al*. [19], Wahyudi *et al*. [20], and Yoelinda *et al*. [21], with fertility rates based on an S/C of 1.52, which is satisfactory. However, in several studies on sperm kinematics that are similar to this study, many different fertility levels were still found [22, 23]. Therefore, a more accurate assessment of a bull’s fertility ability is needed.

A method has been developed to predict bull fertility using protein-containing seminal plasma [9]. Several studies have reported positive results on fertility levels [24, 25]. In seminal plasma, many proteins have reproductive functions, particularly in supporting the movement of spermatozoa. The results of this study showed that the molecular weight distribution of proteins in bull seminal plasma collected from Bali-polled bulls ranged from 13 to 70 kDa. GO annotation was used to ascertain the biological processes, cellular localization, and molecular functions of proteins specific to the seminal plasma of Bali-polled bulls (Figure 1). Reductions in sperm motility are frequently linked to infertility in bulls with sperm kinematics.

Consequently, it is necessary to identify the proteins of the Bali-polled bull to support sperm kinematics. We found 6 proteins in the seminal plasma associated with motility of sperm (Table 2). Proteins such as TEX101, BSP1, PRSS55, BSP3, SPADH2, and TPPP2 play a role in sperm motility; some of these proteins interact with each other to carry out their functions, including TEX101, BSP1, BSP3, and SPADH2 (Figure 3).

TEX101 functions as a cellular component of the plasma membrane raft; however, it is also used in the biological process for the regulation of flagellated sperm motility and binding of sperm to the zona pellucida. It has been determined that the germ-cell-specific protein TEX101 is necessary for the reproduction of males and acts as a cell surface chaperone during the maturation of proteins necessary for sperm motility and sperm-oocyte engagement [26]. BSP1 and BSP3 have cellular functions in cell surface and extracellular space; however, they are involved in biological processes for phospholipid efflux, positive regulation of sperm capacitation, single fertilization, and sperm capacitation. BSP1 is linked to sperm motility and aids protein building, wrapping, and transportation [7, 27]. According to Iskandar *et al*. [11], the BSP1 protein, expressed at high concentrations in bovine seminal plasma, improves sperm motility by activating or boosting Ca^2+^-ATPase activity. BSP3 is a sperm protein binder involved in sperm capacitation and fertilization [28]. SPADH2 functions in the extracellular portion of cells but in the biological process of single fertilization. According to Somashekar *et al*. [29], sperm with high fertility had higher levels of SPADH2. Positive relationships between SPADH2 and freezability [30] and frozen semen fertility [31] have been observed, suggesting that SPADH2 may have a protective effect against oxidative stress.

PRSS55 interacted with RCBTB2, PDILT, TEX37, TEX55, and TSSK4 (Figure 3c). PRSS55 has a function in cellular components of acrosomal vesicles and plasma membranes; however, biological processes have the same function as TEXT101 and proteolysis. Shang *et al*. [32] and Kobayashi *et al*. [33] discovered that PRSS55 deficiency disrupted some sperm functions, such as sperm-uterotubal junction movement and sperm-zona pellucida binding), which reduced fertility. PRSS55 was determined to be required for male reproduction. According to Zhu *et al*. [34], sperm tail injury and aberrant extension are observed in patients lacking PRSS55. TPPP2 interacted with NME8, SLIT3, PPP2R2B, NTN3, and OAZ3 (Figure 3f). TPPP2 plays a role in the cytosol and flagellum of sperm, contributing to various biological processes essential for sperm function and development. These processes include spermatogenesis, the formation of microtubule bundles, microtubule polymerization, positive regulation of protein polymerization, and motility of flagellated sperm. Gacem *et al*. [35] found that TPPP2 was expressed in high-fertility bulls. According to Zhu *et al*. [36], sperm motility is substantially reduced, TPPP2 is suppressed, and sperm ATP content is significantly lower.

Based on this discussion, 6 proteins in the seminal plasma are linked with sperm motility function, including TEX101, BSP1, PRSS55, BSP3, SPADH2, and TPPP2, which can be used as fertility biomarker candidates to be an additional parameter in bull selection.

## CONCLUSION

This study presents a robust evaluation of the reproductive performance of Bali-polled bulls, focusing on sperm kinematics and seminal plasma proteomics. The findings highlight that post-thaw sperm kinematics, including parameters such as velocity and linearity, exhibited satisfactory fertility outcomes with a S/C of 1.52. Proteomic analysis identified 138 seminal plasma proteins, among which six (TEX101, BSP1, PRSS55, BSP3, SPADH2, and TPPP2) were closely associated with sperm motility and fertility. These proteins demonstrate significant biological roles in sperm capacitation, motility regulation, and fertilization, making them promising biomarkers for bull fertility assessment.

The strength of this study lies in its comprehensive and integrative approach combining advanced sperm kinematic evaluation using CASA and detailed proteomic analysis through LC-MS/MS. This dual approach provides a deeper understanding of the molecular mechanisms underlying Bali-polled bull fertility, offering valuable insights for enhancing breeding strategies.

However, the study has limitations. The sample size was relatively small, focusing on five Bali-polled bulls, and no comparison with infertile or low-fertility bulls was conducted. In addition, the absence of 2D-SDS-PAGE constrained the molecular weight profiling accuracy of seminal plasma proteins. These limitations highlight the need for further studies involving larger sample sizes, comparative analysis with infertile bulls, and the inclusion of advanced proteomic techniques.

Future research should explore the application of these biomarkers in field conditions to validate their effectiveness across diverse populations. Investigations into the functional roles of identified proteins in fertility mechanisms and their potential manipulation could pave the way for novel fertility enhancement strategies. Furthermore, integrating genomic and proteomic approaches may provide a more comprehensive framework for improving the reproductive efficiency of Bali-polled bulls and other local breeds.

## AUTHORS’ CONTRIBUTIONS

AMD, SS, HH, MY, ALT, and MIAD: Conceived, designed, and coordinated the study. TPP, SS, PL, and LA: Principal investigators. AMD, TM, HI, and AN: Designed data collection tools. AMD, MY, ALT, and MIAD: Supervised field sampling, data collection, laboratory work, and data entry. AMD, SyS, HH, TM, HI, AN, and AB: Statistical analysis and interpretation and drafted the manuscript. All authors have read and approved the final version of the manuscript.
